# Predictors and responses to varying durations of BTK inhibitor bridging therapy before anti-CD19 CAR-T cell therapy in patients with relapsed/refractory DLBCL

**DOI:** 10.3389/fimmu.2026.1674235

**Published:** 2026-02-06

**Authors:** Jia Wang, Rui Cui, Yao Qi, Juan Mu, Xin Li, Qing Li, Qi Deng

**Affiliations:** Department of Hematology, Tianjin First Central Hospital, School of Medicine, Nankai University, Tianjin, China

**Keywords:** bridging therapy, BTK inhibitors, chimeric antigen receptor (CAR), diffuse large B-cell lymphoma, nicotinamide phosphoribosyl transferase, programmed cell death 1

## Abstract

**Background:**

Anti-CD19 chimeric antigen receptor (CAR)-T cell therapy has demonstrated clinical potential in treating relapsed or refractory (R/R) diffuse large B-cell lymphoma (DLBCL); however, enhancing its therapeutic efficacy remains a significant challenge. To this end, bridging therapy with Brutonyg tyrosine kinase inhibitors (BTKi), such as ibrutinib or zanubrutinib, is being investigated as a strategy to improve treatment outcomes.

**Patients and methods:**

In this retrospective analysis, we assessed the impact of different durations of BTKi bridging therapy prior to anti-CD19 CAR-T cell infusion in 33 patients with R/R DLBCL. Patients meeting predefined eligibility criteria, including the presence of at least one high-risk prognostic factor. These 33 patients were stratified into two groups based on the duration of BTKi exposure: ≥2 months versus <2 months.

**Results:**

The R/R DLBCL patients receiving BTKi for ≥2 months demonstrated a higher overall response rate than the patients receiving BTKi for <2 months. There was no statistically significant differences in progression free survival (PFS) or overall survival (OS) between the two groups. Exploratory analyses suggested potential biomarkers for BTKi bridging efficacy, including modulation of nicotinamide phosphoribosyltransferase (NAMPT) and programmed cell death protein 1 (PD-1).

**Conclusions:**

Prolonged BTKi bridging might improve the overall response to CAR-T cell therapy in patients with R/R DLBCL, despite the initial disparities. However, the risk of hematological toxicity associated with extended BTKi use requires attention. Further investigations are essential to validate and translate these observations into clinical practice, thus highlighting the need for further research in this area.

**Clinical Trial Registration:**

https://www.chictr.org.cn/showproj.aspx?proj=33185, ChiCTR1800019622

## 1 Introduction

Anti-CD19 chimeric antigen receptor (CAR) T-cell therapy has presented significant therapeutic efficacy in patients with relapsed/refractory (R/R) diffuse large B-cell lymphoma (DLBCL), high-grade B-cell lymphoma, mantle cell lymphoma (MCL), and follicular lymphoma (FL) (1–6). Despite an overall response rate (ORR) of 83% and a complete response (CR) rate of 58% in patients in the ZUMA-1 trial, the two-year progression-free survival (PFS) after CAR-T-cell infusion is only 39% (4, 7). Consequently, patients who are unresponsive to this therapy still face disease progression and mortality, which underscores the importance of enhancing the efficacy of anti-CD19 CAR-T cell therapy in patients with R/R DLBCL.

The tumor microenvironment (TME) of lymphoma is one of the important factors for drug resistance (8), and poor TME of lymphoma induces drug resistance to CAR-T (9, 10). The results of ZUMA-1 and ZUMA-7 studies showed that the adverse TME characteristics of R/R LBCL induced drug resistance in clinical CAR-T therapy, especially high-level polarization of M2 macrophages were associated with CAR-T resistance (11–13). Prior studies have indicated that BTKi therapy might downregulate M2 polarization in patients with CLL (14), whereas M2 macrophages inhibit the cytotoxic activity of CAR-T cells against lymphoma cells (13, 15). Nicotinamide phosphoribosyltransferase (NAMPT) plays the role of a rate-limiting enzyme in the biosynthesis of nicotinamide adenine dinucleotide and regulates the tumor microenvironment from a metabolic perspective. NAMPT is elevated in tumors such as lymphoma, promoting the upregulation of M2 polarization in macrophages (16, 17). Therefore, could BTKi reverse the resistance of lymphoma cells to CAR-T cells, thereby enhancing the efficacy of anti-CD19 CAR-T cell therapy, and could NAMPT serve as a more convenient biomarker for down-regulation of M2 polarization? It awaits further clinical exploration.

Previously, we reported the combined efficacy of BTKi and anti-CD19 CAR-T cell therapy *in vitro*, in murine models (18), and in clinical trials (19–21). Notably, the use of BTKis as a bridging therapy prior to anti-CD19 CAR-T cell therapy has led to satisfactory clinical outcomes (19–21). Based on our previous findings, we selected BTKi as a bridging therapy before anti-CD19 CAR-T cell therapy in patients with R/R DLBCL to increase the efficacy of the CAR-T cell therapy. This study aimed to evaluate the efficacy and side effects of varying durations of BTKis as a bridging therapy before anti-CD19 CAR-T cell therapy in patients with R/R DLBCL. Additionally, we sought to identify the clinical predictors of the efficacy of BTKi bridging therapy before anti-CD19 CAR-T cell therapy.

## 2 Materials and methods

### 2.1 Patients enrolled in our study

In this study, we enrolled a cohort of 33 patients diagnosed with R/R DLBCL who presented with at least one high-risk factor for poor prognosis. The enrollment period spanned from July 2019 to March 2022, and all patients underwent BTKi bridging therapy before anti-CD19 CAR-T cell therapy. The high-risk factors for poor prognosis included high tumor burden (defined as at least one tumor with a maximum diameter of ≥ 7.5 cm), Richter transformation to large B-cell lymphoma, the presence of double-hit lymphoma or expression, *TP53* gene deletion/mutation, or the presence of two or more extranodal lesions. The cut-off date for our study was December 31, 2025, and follow-up was extended from the day of anti-CD19 CAR-T cell infusion to the cut-off date or date of death.

### 2.2 BTKi bridging therapy

BTKi bridging therapy, a crucial component of our treatment protocol, involves the administration of ibrutinib or zanubrutinib for durations that range from one-four months prior to the initiation of anti-CD19 CAR-T-cell therapy. The dosage regimens of ibrutinib (420 mg once daily; Xian Janssen Pharmaceutical Ltd.) and zanubrutinib (160 mg twice daily; BeiGene Pharmaceutical Corporation) were standardized for this study. BTKi administration was discontinued in patients with severe hematological toxicity or infectious complications during the bridging period. An assessment of the response to BTKi bridging therapy was performed for all patients with R/R DLBCL before initiating anti-CD19 CAR-T cell therapy. Patients who presented an objective response rate (ORR) following BTKi bridging therapy were allowed to forego subsequent anti-CD19 CAR-T cell infusion.

### 2.3 Grouping method of BTKi bridging therapy

Patients were grouped according to the duration of BTKi bridging therapy and categorized into two groups: those receiving ≥2 months of BTKi therapy and those receiving <2 months. Additionally, patients were stratified based on Richter transformation status into two primary groups: DLBCL and Richter. Patients were subsequently further subdivided into ≥2 months + Richter and <2 months + DLBCL groups on the basis of their respective BTKi duration and Richter transformation status.

The number of patients with DLBCL who received ≥2 months of BTKi bridging therapy and patients with Richter DLBCL who received <2 months of BTKi bridging therapy were notably limited in our study cohort; therefore, these subgroup analyses were not feasible.

### 2.4 Generation of anti-CD19 CAR-T cells

The peripheral blood mononuclear cells (PBMCs) were collected from all patients with R/R DLBCL and isolated by Ficoll density gradient centrifugation. CD3+ T cells were selected by CD3 microbeads (Miltenyi Biotec, Inc., Cambridge, MA, USA) from PBMCs, stimulated by anti-CD3/anti-CD28 mAb-coated Human T-Expander beads (Cat. no. 11141D; Thermo Fisher Scientific, Inc., Waltham, MA, USA) and cultured in T-cell medium X-Vivo 15 (Lonza Group, Ltd., Basel, Switzerland) supplemented with 250 IU/mL interleukin-2 (IL-2; Proluekin; Novartis International AG, Basel, Switzerland). All the CD3+ T cells (2-3x10^6^) were transduced with a lentiviral vector encoding humanized CD19 CAR constructs (10µg, lenti-CD19-2rd-CAR; Shanghai Genbase Biotechnology Co., Ltd. Shanghai, CHINA) and cultured in media containing recombinant human IL-2 (250 U/ml). On the 14-16th day of cultivation, transduction efficiencies of anti-CD19-CAR were analyzed by flow cytometry (FCM) (Shanghai Genbase Biotechnology Co., Ltd. Shanghai, CHINA).

### 2.5 Anti-CD19 CAR-T-cell therapy

Consent for anti-CD19 CAR-T cell therapy was obtained from all 33 patients with R/R DLBCL (*ChiCTR1800019622*). Approval was obtained from the Ethics Committee of the Tianjin First Central Hospital. Lymphodepleting chemotherapy consisting of fludarabine (30 mg/m^2^/day) and cyclophosphamide (400 mg/m^2^/day) was administered from days -4 to -2. The patients received an anti-CD19 CAR-T-cell infusion dose of 2 × 10^6^ cells/kg on day 0 in our study.

Following CAR-T cell infusion, patients received BTKi therapy as maintenance treatment. Discontinuation of BTKi therapy was considered in patients with severe hematological toxicity, infectious diseases, or other adverse effects during maintenance therapy. In patients with complete remission (CR) following CAR-T cell therapy, BTKi therapy was continued for 6-12 months or until the disease progressed. In the event of disease progression following CAR-T cell therapy, BTKi therapy was discontinued.

### 2.6 Expression levels of anti-CD19 CAR-T cells, interleukin-6, programmed cell death 1 and NAMPT

The expression of anti-CD19 CAR-T cells and PD-1 in peripheral blood CD3^+^ T cells was assessed using FCM. The percentage of CD19 CAR-T cell expression in CD3+ T cells were detected by a flow cytometry antibody (PE-CD19 CAR, Shanghai Genbase Biotechnology Co., Ltd. Shanghai, CHINA and FITC-CD3 (145-2C11), BD Biosciences, San Jose, CA, USA), and the expression of PD-1 in CD3+ T cells were detected by a flow cytometry antibody (APC-PD-1 (29F), BD Biosciences, San Jose, CA, USA). FCM plots and gating strategies used to identify lymphocytes: single and viable cells, followed by the selection of CD45 + leukocytes. Then it was followed by the selection of CD3+ T cells, CD3+ CD19 CAR T cells and CD3+ PD-1+ T cells. Stained cells were determined by Beckman Coulter CytoFLEX S flow cytometer (Beckman coulter, United States). The expression of anti-CD19 CAR-T cells in CD3+ T cells were assessed on days 0, 7, 14, 21, and 28 of anti-CD19 CAR-T cell therapy. The expression of PD-1 in CD3^+^ T cells was observed before and after BTKi bridging therapy in the different groups.

Serum NAMPT levels were quantified using an enzyme-linked immunosorbent assay (ELISA), and IL-6 levels were also determined by ELISA. NAMPT concentrations in serum were detected by ELISA kit (Cat. No. CSB-E08940h, Cusabio, Wuhan, China) according to manufacturer’s instructions, and IL-6 levels in serum were detected by ELISA kit (Cat. No. EHC007, NeoBioscience Technology Co.,Ltd, Shenzhen, China). The expression of IL-6 levels in serum were assessed on days 0, 7, 14, 21, and 28 of CD19 CAR-T cell therapy. The expression of NAMPT in serum was observed before and after BTKi bridging therapy in the different groups.

### 2.7 Response criteria

The response to BTKi bridging therapy was assessed prior to anti-CD19 CAR-T cell therapy, and the response to anti-CD19 CAR-T cell therapy was evaluated two months post infusion. The response evaluation was conducted using positron emission tomography-computed tomography or computed tomography. The efficacy assessment was based on the revised Lugano Criteria (22). The primary endpoint was the ORR, which encompassed CR and partial remission (PR), whereas the secondary endpoints were PFS and overall survival (OS). Stable disease (SD) and progressive disease (PD) were defined according to revised Lugano criteria.

### 2.8 Adverse events

Cytokine release syndrome (CRS) and immune effector cell-associated neurotoxic syndrome (ICANS) were graded using a scale developed by the American Society of Transplantation and Cell Therapy (23). Complete blood counts and differentials were graded according to the definitions of the NCI Common Terminology Criteria for Adverse Events, version 5.0 (2017).

### 2.9 Statistical analysis

Normality testing and descriptive statistics: Distribution of continuous variables was assessed using the Shapiro-Wilk test (n<30) or Kolmogorov-Smirnov test (n≥30). Normally distributed data are presented as means ± standard deviation (SD), while non-normally distributed data are presented as median (interquartile range, IQR).

Comparative analyses: For continuous variables, Student’s t-test was used for normally distributed data with homogeneity of variance (confirmed by Levene’s test), and Welch’s t-test for normal data with unequal variances. For non-normal data, the Mann-Whitney U test (non-parametric equivalent to Wilcoxon rank-sum) was applied. Categorical variables were compared using Fisher’s exact test for 2×2 tables with expected cell counts <5 or total n<20; otherwise, chi-square test was used. McNemar’s test was applied for paired categorical data (e.g., response before/after therapy).

Survival analysis: OS and PFS were calculated from CAR-T infusion to death or last follow-up using the Kaplan-Meier method, with differences between groups assessed by log-rank test. Cox proportional hazards models were constructed to estimate hazard ratios (HR) with 95% confidence intervals (CI) after confirming proportional hazards assumptions via Schoenfeld residuals.

Correlative analyses: Associations between continuous variables were assessed by Pearson (normal data) or Spearman (non-normal data) correlation coefficients. All statistical tests were two-sided, and significance was defined as P < 0.05. Analyses were performed using SPSS version 19.0 (IBM Corp., Armonk, NY) and GraphPad Prism version 8.0 (GraphPad Software, San Diego, CA).

## 3 Results

### 3.1 Baseline characteristics

All thirty-three patients with R/R DLBCL exhibited at least one high-risk prognostic factor before receiving anti-CD19 CAR-T cell therapy. In addition to demographic factors such as age and sex, disease characteristics such as Ann Arbor stage, tumor burden, *TP53* gene alterations, Richter transformation, prior treatment history, bridging therapy with different BTKis (ibrutinib/zanubrutinib), baseline International Prognostic Index (IPI) scores, double hit/expression, and the presence of more than one extranodal lesion were worse in the ≥hesenod group than in the <2-month group (**Table 1**).

**Table 1:** Comparison of baseline characteristics between the two groups.

Characteristics	≥2 mons group	<2 mons group	*P* value
n	16	15	
age, mean ± sd	53.69 ± 12.98	54.47 ± 14.08	0.874
sex, n (%)			0.252
Female	3 (18.8%)	6 (40.0%)	
Male	13 (81.3%)	9 (60.0%)	
IPI, n (%)			< 0.001
5	4 (25.0%)	0 (0%)	
4	10 (62.5%)	2 (13.3%)	
3 2	2 (12.5%) 0 (0%)	7 (46.7%) 6 (40.0%)	
Ann Arbor stage, n (%)			0.482
3	7 (43.8%)	9 (60.0%)	
4	7 (43.8%)	6 (40.0%)	
2	2 (12.5%)	0 (0%)	
High tumor load, n (%)			1.000
yes	8 (50.0%)	7 (46.7%)	
no	8 (50.0%)	8 (53.3%)	
Double hit/expression, n (%)			0.011
yes	11 (68.8%)	3 (20.0%)	
no	5 (31.3%)	12 (80.0%)	
TP53 gene deleted/mutated, n (%)			1.000
yes	5 (31.3%)	5 (33.3%)	
no	11 (68.8%)	10 (66.7%)	
Richter transformation, n (%)			0.479
yes	9 (56.3%)	6 (40.0%)	
no	7 (43.8%)	9 (60.0%)	
More than 1 Extranodal lesion, n (%)			0.009
yes	10 (62.5%)	2 (13.3%)	
no	6 (37.5%)	13 (86.7%)	
Lines of prior therapy, n (%)			1.000
6	3 (18.8%)	2 (13.3%)	
5	3 (18.8%)	3 (20.0%)	
4	4 (25.0%)	4 (26.7%)	
3	4 (25.0%)	5 (33.3%)	
2	2 (12.5%)	1 (6.7%)	
Disease state, n (%)			0.273
Relapsed	4 (25.0%)	7 (46.7%)	
Refractory	12 (75.0%)	8 (53.3%)	
Bridging therapy, n (%)			0.149
Ibrutinib	7 (43.8%)	11 (73.3%)	
Zanubrutinib	9 (56.3%)	4 (26.7%)	

### 3.2 Transduction efficiency of anti-CD19 CAR and the infusion dose

The mean transduction efficiency of anti-CD19 CAR in all thirty-three enrolled patients was 42.84 ± 6.11%. Except for two patients who did not receive anti-CD19 CAR-T-cell infusion due to disease progression during BTKi bridging therapy, the remaining thirty-one patients received an intravenous infusion of 2.07 ± 0.42 × 10^6^ cells/kg autologous anti-CD19 CAR-T cells on the day of infusion.

### 3.3 Clinical responses to BTKi bridging therapy and anti-CD19 CAR-T-cell therapy

Only one patient in the ≥h months group developed grade 4 thrombocytopenia that lasted for two days during CAR-T-cell therapy and recovered without requiring treatment discontinuation. None of the other patients discontinued BTKi bridging therapy or BTKi maintenance therapy because of grade ≥r hematological toxicity, infectious diseases, or other adverse effects.

No patients achieved ORR nor received alternative bridging therapy when the clinical response following BTKi bridging therapy was evaluated. Two patients (Pt 11 and Pt 26) who did not receive CAR-T cell infusion died due to disease progression during BTKi bridging therapy. Both patients were diagnosed with Richter transformation large B-cell lymphoma and underwent BTKi bridging therapy for at least two months.

**Figure 1:**
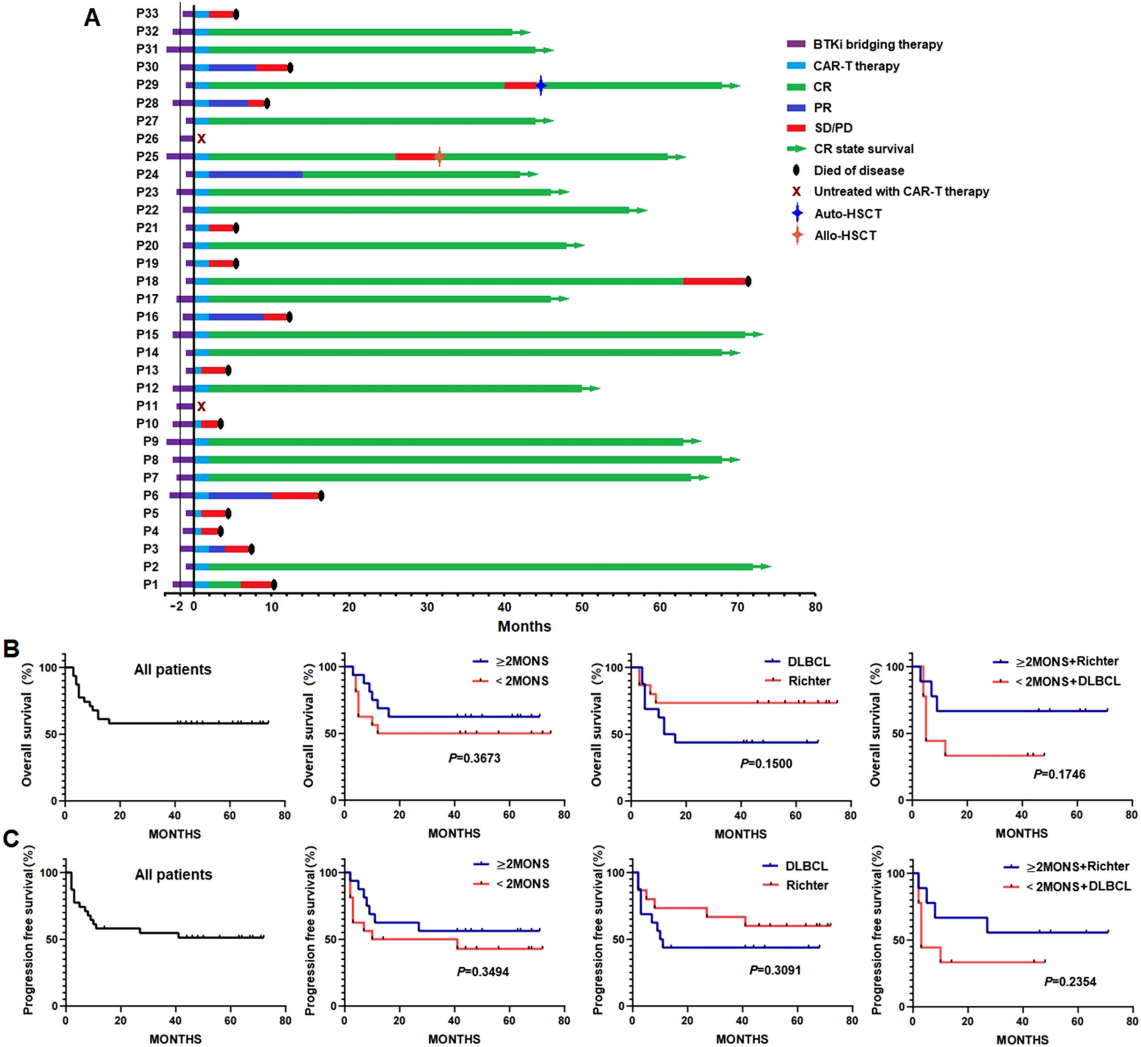
Survival outcomes in different patient groups following anti-CD19 CAR-T cell therapy. **(A)** Clinical responses in all patients, assessed two months post-infusion, including complete response (CR), partial response (PR), stable disease (SD), and progressive disease (PD). **(B, C)** Kaplan–Meier curves for overall survival (OS) and progression-free survival (PFS), showing no significant differences between ≥2 months vs. <2 months BTKi bridging groups, between DLBCL vs. Richter transformation groups, and between ≥2 months + Richter vs. <2 months + DLBCL groups.

The clinical response to anti-CD19 CAR-T cell therapy was evaluated two months post infusion in the treated patients. Among the 31 patients, 18 (58.06%, 18/31) achieved CR, six (19.35%, 6/31) achieved PR, six (19.35%, 6/31) had SD, and one (3.44%, 1/31) had PD only. In the subsequent follow-up, three patients (Pt 18, Pt 25 and Pt 29) who obtained CR experienced disease progression again at 61, 24, and 38 months after being evaluated as CR. All these three patients were in Richter group. Pt 25 and Pt 29 obtained CR again after autologous/allogeneic hematopoietic stem cell transplantation salvage therapy. But the Pt 18 died of a recurrence of lymphoma. All the patients in DLBCL group who were evaluated as CR maintained CR status to the cut-off date. Only one patient (Pt 24) who obtained PR in CAR-T cell therapy achieved CR in BTKi maintenance treatment. All the other five patients who obtained PR died of a recurrence of lymphoma (**Figure 1A**). By the cut-off date, neither OS nor PFS has been achieved. There were no significant differences in OS or PFS between the ≥2 months and <2 months groups (*P*_OS_=0. 3673, *P*_PFS_=0. 3494), between the DLBCL and Richter groups (*P*_OS_=0. 1500, *P*_PFS_=0. 3091), or between the ≥2 months + Richter and <2 months + DLBCL groups (*P*_OS_=0. 1746, *P*_PFS_=0. 2354) (**Figures 1B, C**).

**Figure 2:**
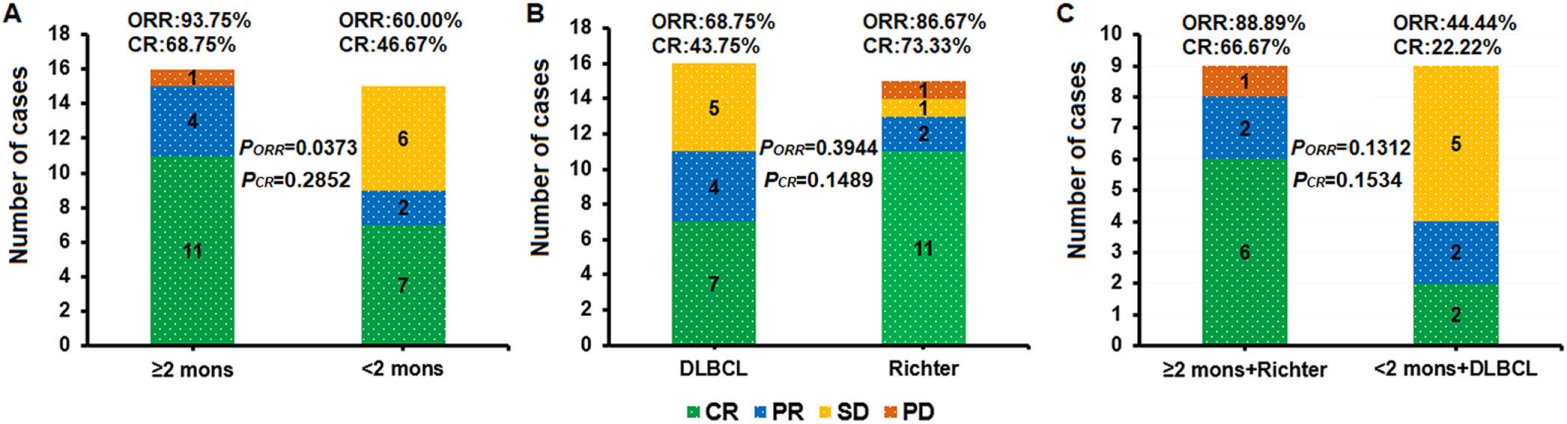
Overall response rate (ORR) and complete response (CR) rates in different lymphoma treatment groups. ORR was significantly higher in the ≥2-month BTKi bridging group compared with the <2-month group (P = 0.0373), while no significant differences were observed between the Richter and DLBCL groups or between the ≥2-month + Richter and <2-month + DLBCL groups. CR rates did not differ significantly among the three comparisons. Bars are color-coded as CR (green), PR (blue), SD (yellow), and PD (orange), with ORR and CR percentages annotated alongside corresponding (PORR) and (PCR) values. **(A)** ≥2 months vs. <2 months BTKi bridging; **(B)** Richter vs. DLBCL; **(C)** ≥2 months + Richter vs. <2 months + DLBCL.

Two months after CAR-T cell infusion in our study, the ORR was 77.42% (24/31) in all the 31 patients. The ORR was 93.75% in the group with a treatment duration of ≥f months and 60.00% in the group with <2 months of treatment (*P* = 0.0373). In the Richter group, the ORR was 86.67% and in the DLBCL group, was 68.75% (*P* = 0.3944). The ORR was 88.89% in the group receiving ≥e months of treatment combined with Richter transformation and 44.44% in the group receiving <2 months of treatment combined with DLBCL (*P* = 0.1312) (**Figures 2A–C**). There was no statistically significant difference in the CR rate among the three groups (*P* = 0.2852, 0.1489, and 0.1534) (**Figures 2A–C**).

### 3.4 Anti-CD19 CAR-T cells and IL-6 expression levels

**Figure 3:**
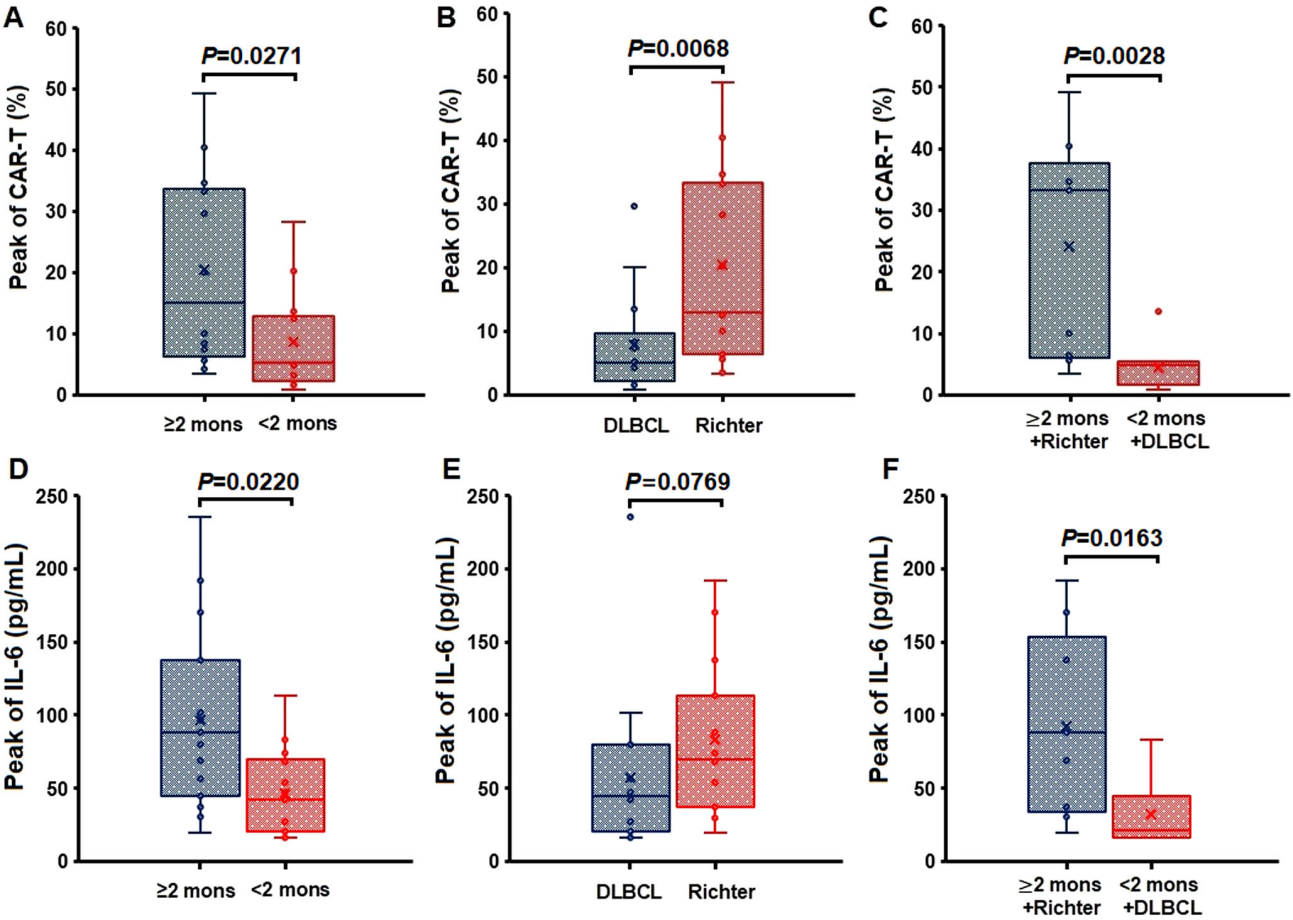
Peak percentages of anti-CD19 CAR-T cells within CD3+ T cells and peak serum IL-6 levels in different patient groups. **(A–C)**: CAR-T cell peaks were higher in the ≥2-month BTKi bridging group (P = 0.0271), the Richter group (P = 0.0068), and the ≥2-month + Richter group (P = 0.0028). **(D–F)**: IL-6 peaks were higher in the ≥2-month BTKi bridging group (P = 0.0220) and the ≥2-month + Richter group (P = 0.0163), with no significant difference between the DLBCL and Richter groups (P = 0.0769). Each panel compares two patient groups, with box plots showing median values, interquartile ranges, and outliers.

For anti-CD19 CAR-T-cell therapy, the peak number of anti-CD19 CAR-T cells was significantly greater in the ≥heaterc group than in the <2-month group (*P* = 0.0271). Similarly, the relevant IL-6 genes were expressed at higher levels in the Richter group than in the DLBCL group (*P* = 0.0068) and in the ≥h months + Richter group than in the <2 months + DLBCL group (*P* = 0.0028) (**Figures 3A–C**).

The peak levels of IL-6 were significantly greater in the ≥h months group than in the <2 months group (*P* = 0.0220) and were also greater in the ≥h months + Richter group than in the <2 months + DLBCL group (*P* = 0.0163). However, no significant difference in the number of IL-6 peaks was observed between the DLBCL group and Richter group (*P* = 0.0769) (**Figures 3D–F**).

### 3.5 NAMPT and PD-1 expression levels in CD3^+^ T cells before and after BTKi bridging therapy

Owing to the lack of serum samples collected before BTKi bridging therapy, NAMPT expression was analyzed in only 22 patients (10 in the ≥h months group and 12 in the <2 months group). The baseline characteristics of these 22 patients were similar to those of the 33 patients (**Supplementary Table 1**).

**Figure 4:**
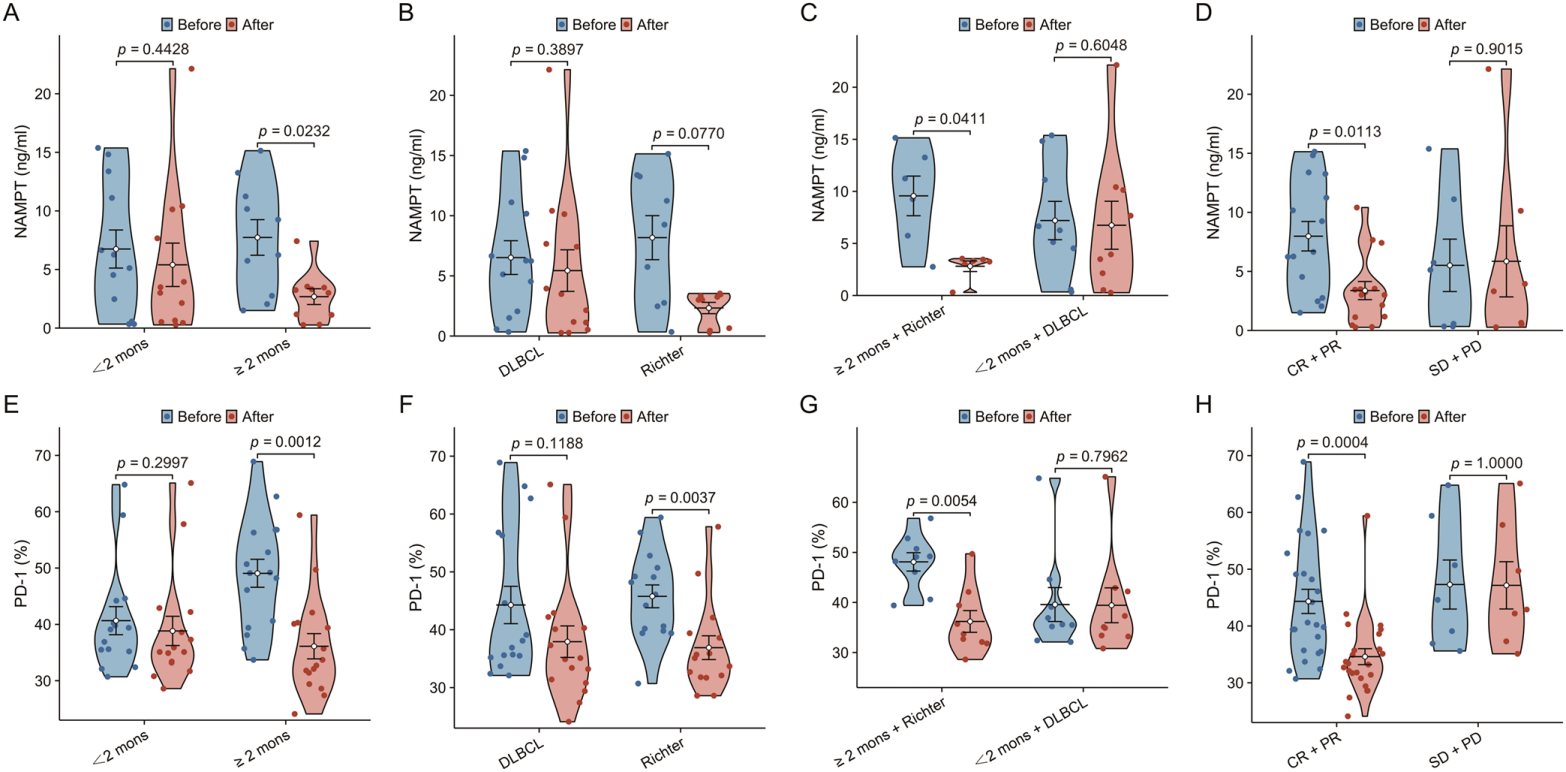
Impact of BTKi bridging therapy on serum nicotinamide phosphoribosyltransferase (NAMPT) and programmed cell death 1 (PD-1) expression in lymphoma patient groups. **(A–D)**: NAMPT levels before and after BTKi therapy, showing significant reductions in the ≥2 months group, the ≥2 months + Richter group, and the CR+PR group; no significant changes in <2 months, DLBCL, Richter, <2 months + DLBCL, and SD+PD groups. **(E–H)**: PD-1 expression in CD3+ T cells before and after BTKi therapy, showing significant reductions in the ≥2 months group, the Richter group, the ≥2 months + Richter group, and the CR+PR group; no significant changes in <2 months, DLBCL, and <2 months + DLBCL groups.

Serum NAMPT levels were assessed before and after the BTKi bridging therapy. The levels were greater before BTKi therapy than after BTKi therapy in the ≥h months group (*P* = 0.0232) and in the ≥h months + Richter group (*P* = 0.0411). No significant differences in NAMPT levels before and after BTKi bridging therapy were observed in the <2 months, DLBCL, Richter, <2 months + DLBCL, and SD+PD groups (*P* = 0.4428, *P* = 0.3897, *P* = 0.0770, and *P* = 0.6048, respectively) (**Figures 4A–C**). Furthermore, NAMPT levels were higher before BTKi therapy than after BTKi therapy in the CR+PR group (P = 0.0113). No statistically significant difference was found before and after BTKi bridging therapy in the SD+PD group (*P* = 0.9015) (**Figure 4D**).

PD-1 expression in CD3^+^ T cells in the peripheral blood was assessed before and after BTKi bridging therapy. PD-1 expression was greater before BTKi therapy than after BTKi therapy in the ≥h months group (*P* = 0.0012), the Richter group (*P* = 0.0037), and the ≥h months + Richter group (*P* = 0.0054). No significant difference in PD-1 expression before or after BTKi bridging therapy was observed in the <2 months, DLBCL, or <2 months + DLBCL groups (*P* = 0.2997, *P* = 0.1188, and *P* = 0.7962, respectively) (**Figures 4E–G**). The level of PD-1 was higher before BTKi therapy than after BTKi therapy in the CR+PR group (*P* = 0.0004). No significant difference was found before and after BTKi bridging therapy in the SD+PD group (*P* = 1.0000) (**Figure 4H**; **Supplementary Figure S1**).

### 3.6 AEs associated with anti-CD19 CAR-T cell therapy

**Figure 5:**
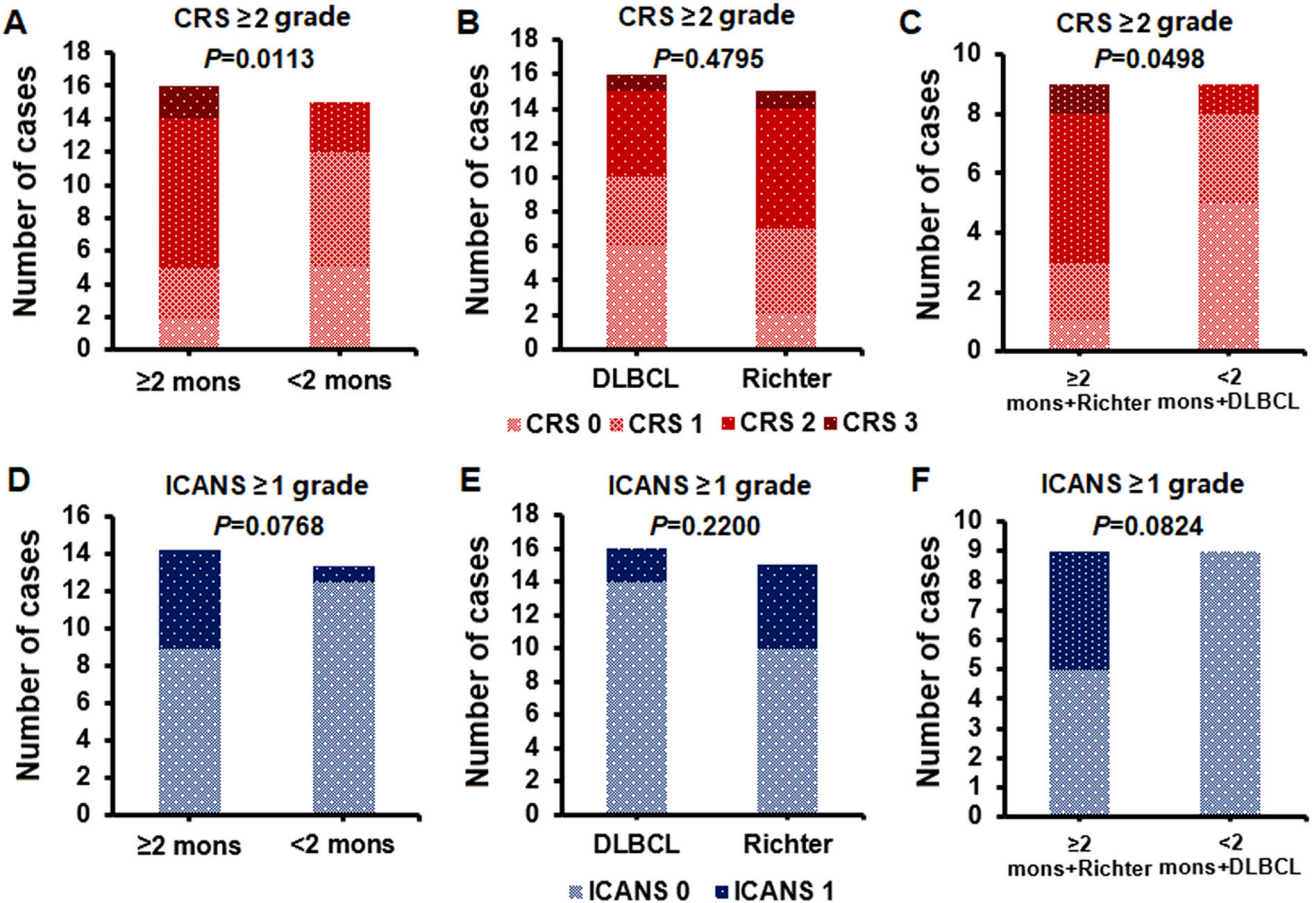
Incidence of cytokine release syndrome (CRS) and immune effector cell-associated neurotoxicity syndrome (ICANS) in different patient groups. **(A–C)**: Incidence of grade ≥2 CRS, higher in the ≥2 months group and the ≥2 months + Richter group; no significant difference between DLBCL and Richter groups. **(D–F)**: Incidence of grade ≥1 ICANS, with no significant differences among ≥2 months vs. <2 months, DLBCL vs. Richter, and ≥2 months + Richter vs. <2 months + DLBCL groups.

All 31 patients with R/R DLBCL recovered from the AEs associated with anti-CD19 CAR-T cell therapy within 15–28 days after CAR-T cell infusion. The incidence of ≥ grade 2 CRS was greater in the ≥h months group than in the <2 months group (*P* = 0.0113) (**Figure 5A**) and in the ≥h months + Richter group than in the <2 months + DLBCL group (*P* = 0.0498) (**Figure 5C**). However, there was no significant difference between the DLBCL group and Richter group (*P* = 0.4795) (**Figure 5B**). Similarly, there was no significant difference in the incidence of ≥ grade 1 ICANS between the ≥h months group and the <2 months group (*P* = 0.0768) (**Figure 5D**), between the DLBCL group and the Richter group (*P* = 0.2200) (**Figure 5E**),or between the ≥h months + Richter group and the <2 months + DLBCL group (*P* = 0.0824) (**Figure 5F**). Only two patients diagnosed with grade 3 CRS received tocilizumab at a dosage of 4 mg/kg/day for one–three days. None of the 31 patients with R/R DLBCL died as a result of any level of CRS or ICANS during anti-CD19 CAR=T cell therapy.

### 3.7 Hematological toxicity in anti-CD19 CAR-T cell therapy

**Figure 6:**
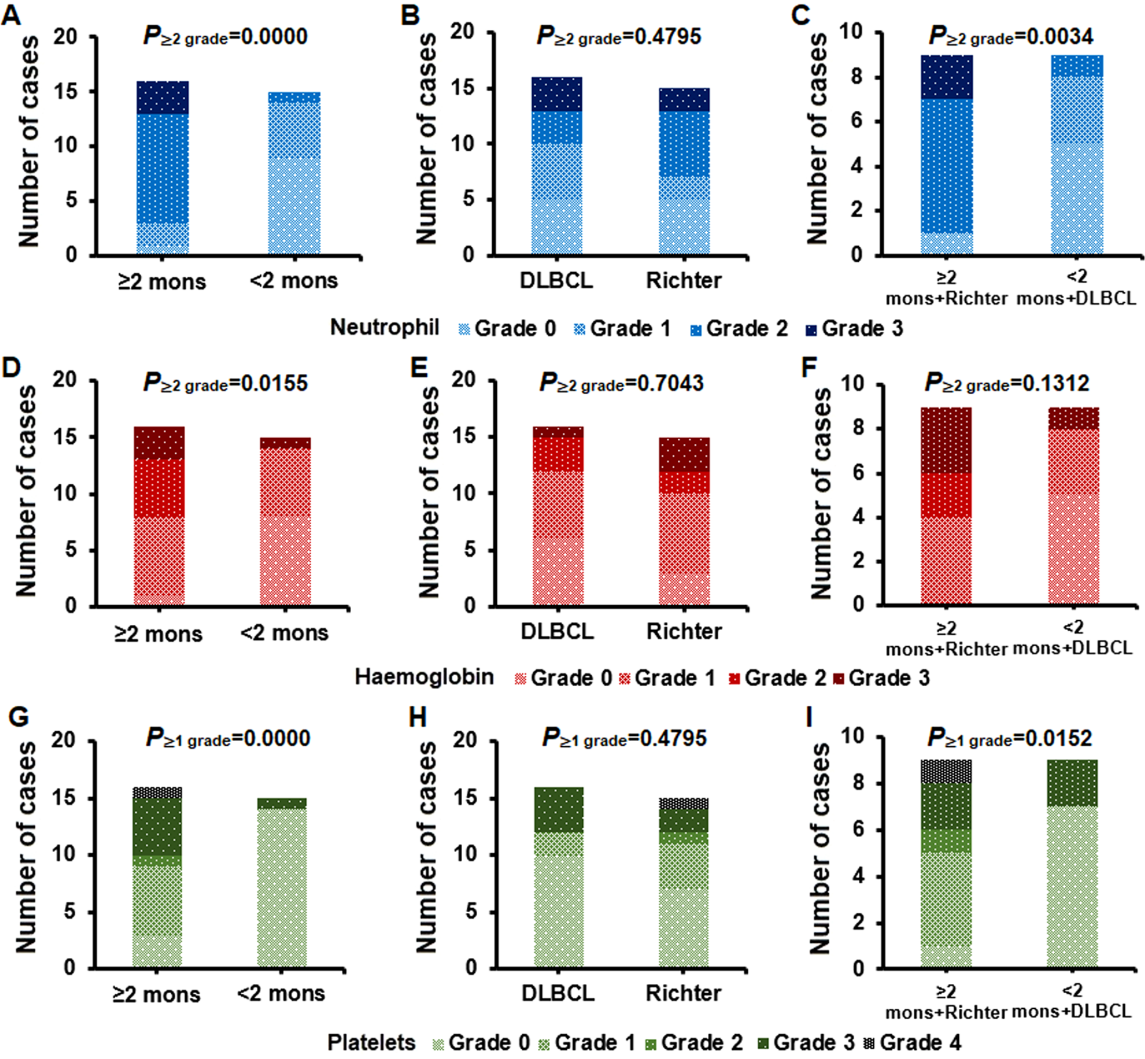
Incidence of hematological toxicities in lymphoma treatment groups. **(A-C)** Grade ≥2 neutropenia, higher in the ≥2 months group and the ≥2 months + Richter group; no significant difference between DLBCL and Richter groups. **(D-F)** Grade ≥2 anemia, higher in the ≥2 months group; no significant differences between DLBCL and Richter groups or between ≥2 months + Richter and <2 months + DLBCL groups. **(G-I)** Grade ≥1 thrombocytopenia, higher in the ≥2 months group and the ≥2 months + Richter group; no significant difference between DLBCL and Richter groups.

The incidence of ≥ grade 2 neutropenia was greater in the ≥h months group than in the <2 months group (*P* < 0.0001) (**Figure 6A**) and in the ≥h months + Richter group than in the <2 months + DLBCL group (*P* = 0.0034) (**Figure 6C**). However, there was no significant difference between the DLBCL group and Richter group (*P* = 0.4795) (**Figure 6B**). Similarly, the incidence of ≥ grade 2 anemia was greater in the ≥h months group than in the <2 months group (*P* = 0.0155) (**Figure 6D**), with no significant difference between the DLBCL group and the Richter group (*P* = 0.7043) (**Figure 6E**) nor between the ≥h months + Richter group and the <2 months + DLBCL group (*P* = 0.1312) (**Figure 6F**). Similarly, the incidence of ≥ grade 1 thrombocytopenia was greater in the ≥h months group than in the <2 months group (*P* = 0.0000) (**Figure 6G**) and in the ≥h months + Richter group than in the <2 months + DLBCL group (*P* = 0.0152) (**Figure 6I**), with no significant difference between the DLBCL group and the Richter group (*P* = 0.4795) (**Figure 6H**). None of the patients in our study died of bacterial or invasive fungal diseases.

## 4 Discussion

Although anti-CD19 CAR-T cell therapy is an effective salvage treatment for patients with R/R B-cell non-Hodgkin lymphoma (B-NHL), there is room for improvement in its efficacy (6, 24, 25). Multiple studies have indicated that ibrutinib may enhance the efficacy of anti-CD19 CAR-T cell therapy in patients with R/R MCL and CLL (2, 26–28). In a study involving 60 R/R patients with MCL previously treated with BTKi therapy, a 93% ORR and 67% CR were achieved using anti-CD19 CAR-T cell therapy (2). The mechanism by which BTKi enhances CAR-T cell efficacy has been elucidated in other studies (26–28). More than three-to-five cycles of ibrutinib therapy has been found to improve the expansion of anti-CD19 CAR-T cells and reduced PD-1 expression on T cells in patients with CLL. Our clinical study further confirmed that ibrutinib downregulates PD-1 expression in peripheral blood T lymphocytes of patients with R/R lymphoma (21). Ibrutinib may inhibit Th2 cell differentiation and promote Th1 cell immune response by inhibiting interleukin-2-inducible T-cell kinase activity. Additionally, the medicine can improve T-cell function by reducing the regulatory T-cell/CD4+ T-cell ratio, decreasing immunosuppressive properties, and enhancing the persistence of activated T cells in patients with R/R CLL through both BTK-dependent and-independent mechanisms (29–33).

Previous studies have reported the ability of ibrutinib therapy to improve TME deficiencies in patients with CLL (34). Analysis of the ZUMA-1 clinical trial involving 51 patients with R/R DLBCL revealed an association between poor TME characteristics and resistance to anti-CD19 CAR-T therapy (11). Our previous study *in vivo* suggested that ibrutinib might enhance the synergistic effects with anti-CD19 CAR-T cells by improving the TME in lymphoma (18). We further explored in clinical practice that BTKi bridging therapy could enhance the efficacy of anti-CD19 CAR-T cell therapy (21), which is also the basis of this study. In our another study, bridging therapy with a BTKi prior to anti-CD19 CAR-T cell therapy reversed clinical responses in patients with R/R FL, and additional poor prognostic factors were identified beyond POD24 (19). In this study, the ORR of ≥2 months BTKi bridging therapy was higher than that of <2 months BTKi bridging therapy in our study. Moreover, although there was no statistical significance, the ORR of group ≥2 months + Richter was 88.89% and that of group <2 months + DLBCL was 44.44%. Could our results suggest that longer BTKi bridging therapy could enhance the efficacy of CAR-T cell therapy, especially for Richter patients? This is consistent with our previous study (19) and is also correlated with the efficacy of BTKi in R/R follicular cell lymphoma (20).

Few studies have compared the efficacy and side effects of various durations of BTKi bridging therapy before anti-CD19 CAR-T cell therapy. It is extremely difficult to determine. In before studies, BTKi bridging therapy might increase the ORR of anti-CD19 CAR-T cell therapy in R/R CLL patients and R/R DLBCL patients with high-risk (35, 36). All the patients received one to three cycles of Zanubrutinib based bridging therapy. So, we chose two months as the dividing point between the two groups. However, few studies have compared the efficacy and side effects of various durations of BTKi bridging therapy before anti-CD19 CAR-T cell therapy. In our study, despite lower baseline IPIs, double-hit lesions/expressions, and more than one extranodal lesion in the group with a duration of ≥f months than in the <2 months group, there were no significant differences in PFS or OS between the two groups. Additionally, the ORR was greater in the ≥h months group. Although there was no disparity in ORRs between the ≥h months + Richter group and <2 months + DLBCL group, the ORRs presented a particular advantage in the Richter and ≥n months + Richter groups. The peaks of anti-CD19 CAR-T cell levels were greater in the ≥h months group, the Richter group, and the ≥h months + Richter group. Our results suggest that prolonging the duration of BTKi bridging therapy prior to anti-CD19 CAR-T cell therapy could improve outcomes in patients with R/R DLBCL with poor baseline characteristics.

Another outcome of our study was the identification of alterations in the expression of NAMPT and PD-1 before and after BTKi bridging therapy. NAMPT serves as a rate-limiting enzyme in nicotinamide adenine dinucleotide biosynthesis, and serum NAMPT levels correlate with the activation and proliferation of tumor cells, including lymphoma cells. NAMPT plays a crucial role in the survival of lymphoma cells, and lymphoma cell-derived NAMPT promotes macrophage M2 polarization (14, 16). M2 macrophage polarization correlates with poor survival in patients with DLBCL, and M2-type macrophages inhibit the cytotoxic effects of CAR-T cells on lymphoma cells (13, 15). Furthermore, BTKi therapy has been found to downregulate M2 macrophage polarization in patients with CLL (14). In this study, we observed that the level of serum NAMPT was greater before BTKi bridging therapy than after BTKi therapy in the CR+PR and ≥2 months groups; however, no difference before and after BTKi bridging therapy was noted between the SD+PD and <2 months groups. We also found that the PD-1 expression level was greater before BTKi bridging therapy than after BTKi therapy in the CR+PR and ≥2 months groups, consistent with the findings of our previous clinical study (21). Our findings also suggest that the downregulation of NAMPT and PD-1 expression may serve as a clinical predictor of the efficacy of BTKi bridging therapy prior to anti-CD19 CAR-T cell therapy. Further expansion of the clinical sample size is necessary to determine the generalizability of our findings.

Notably, the potential side effects of extending the duration of BTKi bridging therapy prior to anti-CD19 CAR-T cell therapy should be considered. The incidence of CRS ≥ grade 2 was greater in the groups with durations ≥u months, particularly in the ≥h months + Richter group; however, there was no significant difference in the incidence of ICANS ≥ grade 1 between groups. Notably, the hematological toxicity associated with BTKi-bridging therapy requires attention. The incidence of neutropenia ≥ grade 2 and thrombocytopenia ≥ grade 1 was greater in the group with a duration of ≥f months. Although none of the patients in our study succumbed to bacterial infections or invasive fungal diseases, implementing infection prevention measures throughout the course of BTKi bridging therapy before anti-CD19 CAR-T cell therapy is crucial for improving patient outcomes.

## 5 Conclusions

Our study revealed that extending the duration of BTKi bridging therapy prior to anti-CD19 CAR-T cell therapy has the potential to enhance outcomes in patients with R/R DLBCL with unfavorable baseline characteristics, such as higher IPI, double hit/expression, and multiple extranodal lesions. Furthermore, to identify the clinical predictors of the efficacy of BTKi bridging therapy, we propose that downregulation of NAMPT and PD-1 expression could serve as predictors. Validation of these findings requires further expansion of the clinical sample size. Moreover, maintaining vigilance against hematological toxicity resulting from the extension of BTKi bridging therapy before anti-CD19 CAR-T cell therapy is essential.

## Data Availability

The raw data supporting the conclusions of this article will be made available by the authors, without undue reservation.
