# Correction: Predictors and responses to varying durations of BTK inhibitor bridging therapy before anti-CD19 CAR-T cell therapy in patients with relapsed/refractory DLBCL

**DOI:** 10.3389/fimmu.2026.1814535

**Published:** 2026-03-09

**Authors:** Jia Wang, Rui Cui, Yao Qi, Juan Mu, Xin Li, Qing Li, Qi Deng

**Affiliations:** Department of Hematology, Tianjin First Central Hospital, School of Medicine, Nankai University, Tianjin, China

**Keywords:** bridging therapy, BTK inhibitors, chimeric antigen receptor (CAR), diffuse large B-cell lymphoma, nicotinamide phosphoribosyl transferase, programmed cell death 1

In the *Abstract*, two typographical errors were identified. In the last sentence of the *Patients and Methods* section, the original version incorrectly stated: “These 33 patients were stratified into two groups based on the duration of BTKi exposure: ≥x months versus <2 months.” In the first sentence of the *Results* section, it incorrectly stated: “The R/R DLBCL patients receiving BTKi for ≥o months demonstrated a higher overall response rate than the patients receiving BTKi for <2 month.”.

The corrected *Abstract* reads: “These 33 patients were stratified into two groups based on the duration of BTKi exposure: ≥2 months versus <2 months,” and “The R/R DLBCL patients receiving BTKi for ≥2 months demonstrated a higher overall response rate than the patients receiving BTKi for <2 months.”

The original version of this article has been updated.

