# Supplementary figures and images for: Predictors and responses to varying durations of BTK inhibitor bridging therapy before anti-CD19 CAR-T cell therapy in patients with relapsed/refractory DLBCL
Source: Front Immunol. 2026 Feb 6;17:1674235. doi: 10.3389/fimmu.2026.1674235 (PMC12920493; doi:10.3389/fimmu.2026.1674235)

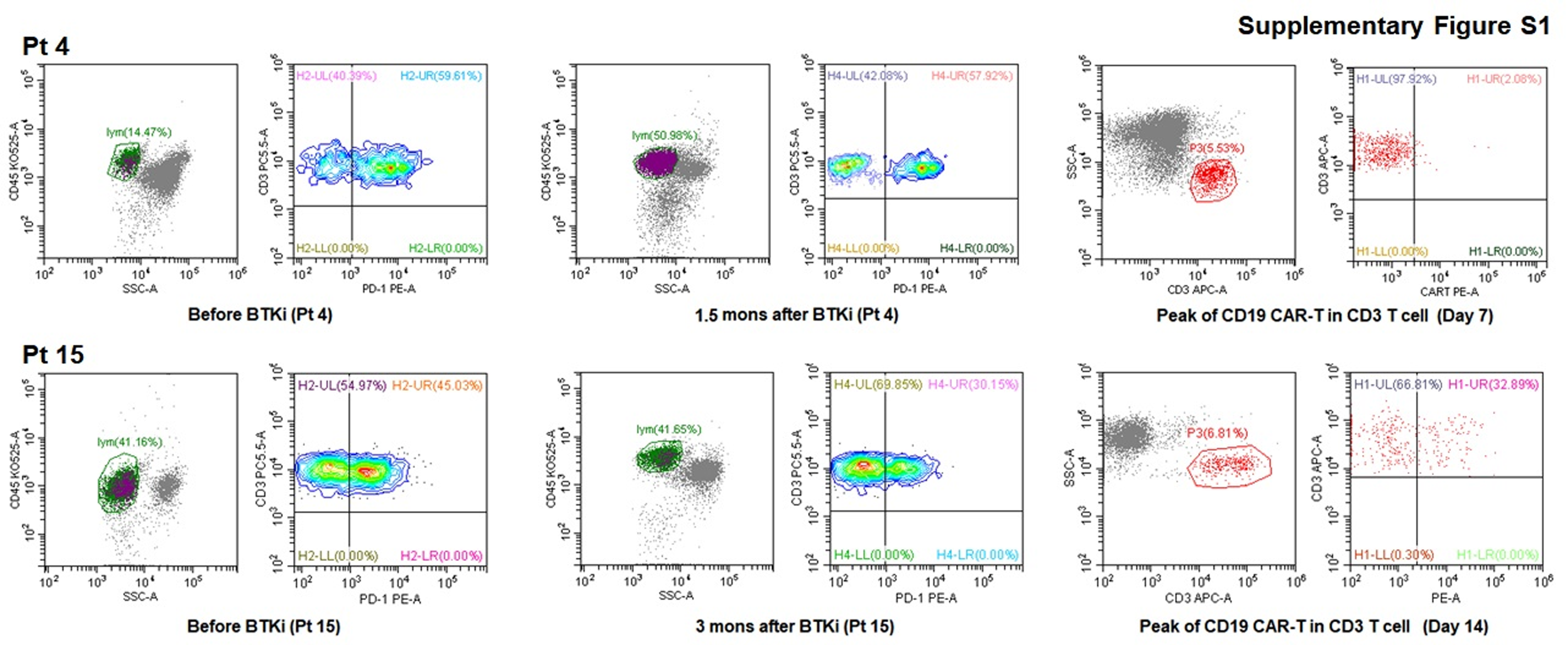

Supplement: Supplementary file 1 [file Image1.tif]
