# Supplementary material for: Predictors and responses to varying durations of BTK inhibitor bridging therapy before anti-CD19 CAR-T cell therapy in patients with relapsed/refractory DLBCL
Source: Front Immunol. 2026 Feb 6;17:1674235. doi: 10.3389/fimmu.2026.1674235 (PMC12920493; doi:10.3389/fimmu.2026.1674235)
Supplement: Supplementary file 2 [file Table1.docx]

**Supplementary Table 1.** Comparison of baseline characteristics between the two groups

| **Characteristics** | **≥2 mons group** | **＜2 mons group** | ***P* value** |
| --- | --- | --- | --- |
| **n** | 10 | 12 |  |
| **age, mean ± sd** | 52.7 ± 15.1 | 53.833 ± 15.3 | 0.864 |
| **sex, n (%)** |  |  | 0.204 |
| **Female** | 2 (9.1%) | 6 (27.3%) |  |
| **Male** | 8 (36.4%) | 6 (27.3%) |  |
| **IPI, n (%)** |  |  | 0.022 |
| **5** | 3 (13.6%) | 0 (0%) |  |
| **4** | 5 (22.7%) | 2 (9.1%) |  |
| **3** | 2 (9.1%) | 7 (31.8%) |  |
| **2** | 0 (0%) | 3 (13.6%) |  |
| **Ann Arbor stage, n (%)** |  |  | 0.069 |
| **4** | 6 (27.3%) | 5 (22.7%) |  |
| **3** | 2 (9.1%) | 7 (31.8%) |  |
| **2** | 2 (9.1%) | 0 (0%) |  |
| **High tumor load, n (%)** |  |  | 1.000 |
| **yes** | 5 (22.7%) | 7 (31.8%) |  |
| **no** | 5 (22.7%) | 5 (22.7%) |  |
| **Double hit/expression, n (%)** |  |  | 0.006 |
| **yes** | 7 (31.8%) | 1 (4.5%) |  |
| **no** | 3 (13.6%) | 11 (50%) |  |
| **TP53 gene deleted/mutated, n (%)** |  |  | 1.000 |
| **yes** | 3 (13.6%) | 4 (18.2%) |  |
| **no** | 7 (31.8%) | 8 (36.4%) |  |
| **Richter transformation, n (%)** |  |  | 0.192 |
| **yes** | 6 (27.3%) | 3 (13.6%) |  |
| **no** | 4 (18.2%) | 9 (40.9%) |  |
| **More than1 Extranodal lesion, n (%)** |  |  | 0.027 |
| **yes** | 7 (31.8%) | 2 (9.1%) |  |
| **no** | 3 (13.6%) | 10 (45.5%) |  |
| **Prelines of therapy, mean ± sd** | 4.1 ± 1.3703 | 4.3333 ± 1.0731 | 0.659 |
| **Disease state, n (%)** |  |  | 0.204 |
| **Relapsed** | 2 (9.1%) | 6 (27.3%) |  |
| **Refractory** | 8 (36.4%) | 6 (27.3%) |  |
| **Bridging therapy, n (%)** |  |  | 0.378 |
| **Ibrutinib** | 5 (22.7%) | 9 (40.9%) |  |
| **Zanubrutinib** | 5 (22.7%) | 3 (13.6%) |  |

**Figure legends**

**Supplementary Figure S1** The expression of PD-1 before and after BTKi treatment and the peak expression of CAR-T cell in CD3+ T cell.
